# Chronic Pulmonary Silicone Embolism from Breast Augmentation Is Not a Common Finding in Explanted Lungs

**DOI:** 10.1155/2018/2987072

**Published:** 2018-03-15

**Authors:** Jarmanjeet Singh, Hanine Inaty, Sanjay Mukhopadhyay, Atul C. Mehta

**Affiliations:** ^1^Internal Medicine Residency Program, The Cleveland Clinic Foundation, Cleveland, OH, USA; ^2^Respiratory Institute, The Cleveland Clinic Foundation, Cleveland, OH, USA; ^3^Department of Pathology, The Cleveland Clinic Foundation, Cleveland, OH, USA

## Abstract

**Objective:**

Acute pulmonary silicone embolism (APSE) related to subcutaneous silicone injections is a well-known entity. Recently, a few cases of pathologically confirmed chronic pulmonary silicone embolism (CPSE) from breast implants have been reported. The prevalence of CPSE in women with breast augmentation is unknown. This study was done to determine the prevalence of CPSE in female lung transplant recipients with a history of breast augmentation and to determine whether breast augmentation plays a role in chronic lung diseases requiring lung transplantation.

**Methods:**

A retrospective chart review was performed to identify female lung transplant recipients with a history of breast augmentation prior to or at the time of lung transplantation. Ten patients meeting these criteria were identified. The pathologic features of the explanted lungs of these patients were reexamined for CPSE by a board-certified pathologist with expertise in lung transplantation and pulmonary embolism.

**Results:**

Of 1518 lung transplant recipients at Cleveland Clinic, 578 were females. Of 578 females, 10 (1.73%) had history of breast augmentation. A total of 84 H&E-stained slides from the explanted lungs from 10 cases were examined. No pathologic evidence of chronic silicone embolism was seen in any of the 10 cases.

**Conclusions:**

CPSE is not associated with pulmonary disease leading to lung transplantation. Breast augmentation is not a significant contributor to pulmonary disease requiring lung transplantation. Further studies are required to ascertain the prevalence of CPSE in the general breast augmentation populace and to define the relationship between breast augmentation and pulmonary disease.

## 1. Introduction

The concept of breast implantation was introduced in the 18th century by Vincenz Czerny who used a lipoma for breast augmentation [1]. Since then, breast implantation has evolved considerably, and breast implants are composed of an outer elastomer shell of silicone and an inner filler, which can be either silicone gel or saline. Silicone used in breast implants is polydimethylsiloxane (PDMS) and is considered safe [2]. Acute pulmonary silicone embolism (APSE) from cosmetic silicone injections is well known to cause lung disease [3–8]. Recently, cases of chronic pulmonary silicone embolism (CPSE) related to silicone and saline breast implants causing dyspnea and pulmonary infiltrates have been reported [9–13] (Table 1). CPSE is embolization of silicone to the lungs in women with breast implantation but with no history of subcutaneous silicone injection. These cases made us wonder whether CPSE is an underrecognized contributor to chronic pulmonary ailments. This study was done to determine the prevalence of CPSE in female lung transplant recipients with a history of breast augmentation and to determine whether breast augmentation plays a role in chronic lung diseases requiring lung transplantation.

## 2. Methods

Medical records of the lung transplant population at Cleveland Clinic (July 1990–May 2016) were reviewed retrospectively to identify female lung transplant recipients who had undergone breast implantation prior to the lung transplantation. Data was gathered for the following variables: demographics, current status of patient (alive or deceased), smoking history, type of breast implant (saline or silicone), duration of breast augmentation prior to transplantation, history of implant rupture, unilateral versus bilateral lung transplantation, primary diagnosis leading to lung transplantation, radiologic and pathologic findings, and survival of candidate after transplantation.

### 2.1. Pathologic Examination

Histopathologic examination of explanted lungs was performed by a fellowship-trained pulmonary pathologist with expertise in pulmonary silicone embolism [10, 14]. Standard protocol including analysis of 3 sections (peripheral, mid, and central) of alveolated lung per lobe was used. Additional sections were taken if areas of special interest were identified on gross examination such as a nodule, cyst, and cavity. These sections were reexamined specifically for evidence of silicone embolization within pulmonary blood vessels.

## 3. Results

Of 1518 lung transplant recipients, 578 were females. Of these females, 10 (1.73%) had history of breast augmentation; 3 had silicone implants, 1 had saline implant, and, in 6, the type of implant could not be determined. The mean duration of breast implantation prior to lung transplantation was 29.25 years (4/10). 2 females had evidence of implant rupture on gross examination at the time of surgery. 8 were smokers with a mean of 34.4 pack-years and 2 were nonsmokers. Lung transplantation was unilateral in 7 and bilateral in 3. 8 had emphysema and 2 had bronchiectasis on chest CT. Indication for lung transplantation was chronic obstructive pulmonary disease in 7 patients, and cystic fibrosis, ciliary dyskinesia, and alpha-1 antitrypsin deficiency in one patient each. 4 patients were alive at the time of this study, with a mean age of 66.25 years (range: 59–77). The mean age at death was 52.2 years (range: 21–70). A total of 84 hematoxylin-eosin-stained slides from the explanted lungs (range: 3–23 slides per case; mean: 8.4) were examined for evidence of silicone emboli. None of the slides showed pathologic evidence of silicone embolism (Table 2).

## 4. Discussion

Reported pulmonary complications from silicone breast implants include silicone thorax [15, 16], pleural effusions [17], silicone-related complaints [18–20], siliconosis, autoimmune/inflammatory syndrome induced by adjuvants (ASIA) [21–23], and anaplastic large cell lymphoma [24, 25]. The presumed pathogenesis behind these complications is the inflammatory response elicited by migration of silicone particles into tissues [26]. Silicone particles and silicone-laden macrophages have been demonstrated at distant sites in affected individuals [12, 27, 28].

Prompted by safety concerns, a moratorium was placed on use of silicone breast implants in the United States between 1992 and 2006 [19]. After reapproval from the FDA in 2006, silicone implants regained their popularity. At present, breast implantation is the most common cosmetic surgery in the United States, with approximately 279,143 cosmetic and 106,338 reconstructive breast implantations performed in 2015 alone [29]. The recent discovery of CPSE related to breast implants has rekindled the controversy regarding the safety of breast augmentation.

Since both cosmetic silicone injections and breast implants contain PDMS, acute pulmonary silicone embolism from silicone injections can help us in understanding the disease spectrum of CPSE due to molecular similarity. Clinicopathological findings in APSE include congestion, pneumonitis, diffuse alveolar damage causing adult respiratory distress syndrome (ARDS), and death [3–8]. Schmid et al. showed that acute silicone embolism can have the same critical presentation as fat embolism with a mean Schonfeld score of 9 [8]. Therefore, CPSE also carries the potential of causing grave complications like acute respiratory failure and death. Fortunately, the reported cases of CPSE have presented as a chronic form of lung disease mimicking interstitial lung disease (ILD) [9–13].

In our study, we did not see any evidence of pulmonary silicone embolism from breast implants in the explanted lungs of the lung transplant recipients. Due to small sample size of 10 patients, we cannot entirely exclude the occurrence of CPSE in the studied population but the data is strongly convincing towards lack of association. Moreover, only 10 females (1.73%) had history of breast implantation among a total of 578 female lung transplant recipients, suggesting no significant association between the two. This lack of association does underline the rarity of CPSE.

To our knowledge, this is the first original study that has attempted to identify CPSE in women with breast implants by pathologic analysis of the explanted lungs. We acknowledge that female lung transplant recipients with breast augmentation constitute an extremely small fraction of the general population that undergoes breast augmentation. The absence of CPSE in this small sample should not be generalizable to regular breast implant recipients in general. However, the absence of CPSE in this large female lung transplant population at our hospital shows its rarity and insignificance in lung transplantation epidemiology. Nevertheless, pulmonologists should be aware of this rare but potentially grave complication and should consider CPSE in the differential of pulmonary pathology in patients with breast implants. Larger studies are required to ascertain the extent to which CPSE might cause occult or symptomatic lung disease not necessarily requiring lung transplantation in general breast implant population.

## 5. Summary/Conclusion

Our study did not show any evidence of CPSE in female lung transplant recipients who had undergone breast augmentation prior to lung transplantation. We conclude that breast augmentation is not a significant contributor to pulmonary diseases requiring lung transplantation. Based upon reported cases, CPSE can cause significant pulmonary morbidity. Further studies are required to determine the prevalence of CPSE in the general breast implant population and to define the relationship between breast augmentation, CPSE, and pulmonary diseases.

## Figures and Tables

**Table 1:** Reported cases of chronic pulmonary silicone embolism from the breast implants.

Case	Age/sex	Symptoms	Radiographic finding on CT chest	Type of implant (silicone versus saline)	Histopathology (microscopic and energy-dispersive X-ray analysis, EDX^$^)
1	41/F	Dyspnea	Diffuse GGO^*∗*^	Silicone	(i) Clear, nonbirefringent globules characteristic of silicone emboli (ii) EDX not performed

2	45/F	Dyspnea	Diffuse GGO	Saline	(i) Clear, nonbirefringent globules on microscopy (ii) Energy-dispersive X-ray microanalysis (EDX) showed silicone emboli

3	72/F	Dyspnea	Diffuse GGO and reticular opacities	Silicone	(i) Clear, nonbirefringent globules on microscopy (ii) Energy-dispersive X-ray microanalysis (EDX) showed silicone emboli

4	56/F	Chronic illness	Not available	Silicone	(i) Silicone plaques in the lungs on microscopy (ii) Energy-dispersive X-ray microanalysis (EDX) showed silicone emboli

5	59/F	Dyspnea	Diffuse pulmonary infiltrates and consolidations	Double lumen prosthesis (cohesive gel and saline solution)	(i) Translucent vacuolated globular deposits of silicone

^*∗*^GGO: ground glass opacities; ^$^EDX: energy-dispersive X-ray analysis.

**Table 2:** Radiographic and pathologic findings of the explanted lungs.

Case	Duration of breast implantation before lung transplant (in years)	Radiographic diagnosis before lung transplantation	Number of slides studied	Pathologic diagnosis	Evidence of breast implant rupture at the time of lung transplantation surgery	Silicone emboli in explanted lungs
1	Unknown	Bronchiectasis	9	Bronchiectasis	Not available	Absent
2	Unknown	Emphysema	6	Emphysema	Not available	Absent
3	25	Emphysema	5	Emphysema	Not available	Absent
4	Unknown	Emphysema	6	Emphysema	Not available	Absent
5	Unknown	Emphysema	8	Emphysema	Not available	Absent
6	Unknown	Emphysema	8	Emphysema	Not available	Absent
7	25	Emphysema	3	Emphysema	Intracapsular rupture	Absent
8	Unknown	Bronchiectasis	23	Bronchiectasis	Not available	Absent
9	30	Emphysema	9	Emphysema	Not available	Absent
10	35	Emphysema	7	Emphysema	Focal rupture	Absent
